# Spatial pattern of mortality from breast and cervical cancer in the city of São Paulo

**DOI:** 10.11606/s1518-8787.2020054002447

**Published:** 2020-12-04

**Authors:** Patricia Marques Moralejo Bermudi, Alessandra Cristina Guedes Pellini, Elizabeth Angélica Salinas Rebolledo, Carmen Simone Grilo Diniz, Breno Souza de Aguiar, Adeylson Guimarães Ribeiro, Marcelo Antunes Failla, Oswaldo Santos Baquero, Francisco Chiaravalloti

**Affiliations:** I Universidade de São Paulo Faculdade de Saúde Pública Departamento de Epidemiologia São PauloSP Brasil Universidade de São Paulo. Faculdade de Saúde Pública. Departamento de Epidemiologia. São Paulo, SP, Brasil; II Universidade Nove de Julho Faculdade de Medicina Diretoria de Ciências Médicas São PauloSP Brasil Universidade Nove de Julho. Faculdade de Medicina. Diretoria de Ciências Médicas. São Paulo, SP, Brasil; III Universidade de São Paulo Faculdade de Saúde Pública Programa de Pós-Graduação em Saúde Pública São PauloSP Brasil Universidade de São Paulo. Faculdade de Saúde Pública. Programa de Pós-Graduação em Saúde Pública. São Paulo, SP, Brasil; IV Universidade de São Paulo Faculdade de Saúde Pública Departamento de Saúde São PauloSP Brasil Universidade de São Paulo. Faculdade de Saúde Pública. Departamento de Saúde, Ciclos de Vida e Sociedade. São Paulo, SP, Brasil; V Secretaria Municipal de Saúde de São Paulo Coordenação de Epidemiologia e Informação Gerência de Geoprocessamento e Informações Socioambientais São Paulo,SP Brasil Secretaria Municipal de Saúde de São Paulo. Coordenação de Epidemiologia e Informação. Gerência de Geoprocessamento e Informações Socioambientais. São Paulo, SP, Brasil; VI Hospital de Câncer de Barretos Instituto de Ensino e Pesquisa BarretosSP Brasil Hospital de Câncer de Barretos. Instituto de Ensino e Pesquisa. Barretos, SP, Brasil; VII Universidade de São Paulo Faculdade de Medicina Veterinária e Zootecnia Departamento de Medicina Veterinária Preventiva e Saúde Animal São PauloSP Brasil Universidade de São Paulo. Faculdade de Medicina Veterinária e Zootecnia. Departamento de Medicina Veterinária Preventiva e Saúde Animal. São Paulo, SP, Brasil

**Keywords:** Breast Neoplasms, Uterine Cervical Neoplasms, Spatial Analysis, Socioeconomic Analysis, Mortality

## Abstract

**OBJECTIVE:**

To verify the spatial pattern of mortality from breast and cervical cancer in areas of primary health care, considering socioeconomic conditions.

**METHODS:**

This is an ecological study, from January 2000 to December 2016. The study area is the municipality of São Paulo, Brazil, and its 456 coverage areas of primary health units. Information on deaths of women aged 20 years or over were geocoded according to residence address. We calculated mortality rates, standardized by age, and smoothed by the local empirical Bayesian method, and grouped into three or two years to reduce the random fluctuation of the data. In addition, bivariate global and local Moran indexes were calculated to verify the existence of spatial agglomeration of standardized mortality rates with a domain of socioeconomic condition, elaborated based on the *Índice Paulista de Vulnerabilidade Social* (IPVS – São Paulo Index of Social Vulnerability).

**RESULTS:**

The success rate of geocoding was 98.9%. Mortality from breast cancer, without stratification by time, showed a pattern with higher rates located in central regions with better socioeconomic conditions. It showed a decrease at the end of the period and a change in spatial pattern, with increased mortality in peripheral regions. On the other hand, mortality from cervical cancer remained with the highest rates in peripheral regions with worse socioeconomic conditions, despite being reduced over time.

**CONCLUSION:**

The spatial pattern of mortality from the studied cancers, over time, suggests association with the best socioeconomic conditions of the municipality, either as protection (cervical) or risk (breast). This knowledge may direct resources to prevent and promote health in the territories.

## INTRODUCTION

Breast and cervical cancer are important global public health problems. In Brazil, breast cancer in the female population increased between the estimates of 2018–2019 and 2020–2022, and remained as the most incident cancer, except for non-melanoma skin cancer. Cervical cancer is one position up and is now the third most incident. Both cancers are also important causes of mortality in the country^1^.

In the municipality of São Paulo (MSP), between 2008 and 2012, the mean annual mortality rate from breast cancer, standardized by age, was 16.2 per 100 thousand women-year, and, for cervical cancer, 3.7 deaths per 100 thousand women-year, with a tendency to decrease in the first cancer and stability in the second^4^.

Some studies performed in the MSP, from 1985–1999 and 2010–2012, verified that regions with better socioeconomic conditions had a higher risk of mortality from breast cancer. Regions with worse socioeconomic conditions, on the contrary, indicated a higher risk of mortality from cervical cancer when compared to the other analyzed territories^5,6^.

This study aimed to verify the spatial pattern of mortality from breast and cervical cancer in women aged 20 years or over, living in the MSP, from 2000 to 2016, comparing these cancers and considering the socioeconomic conditions of the municipality, in areas covered by primary health care.

## METHODS

**Figure 1:**
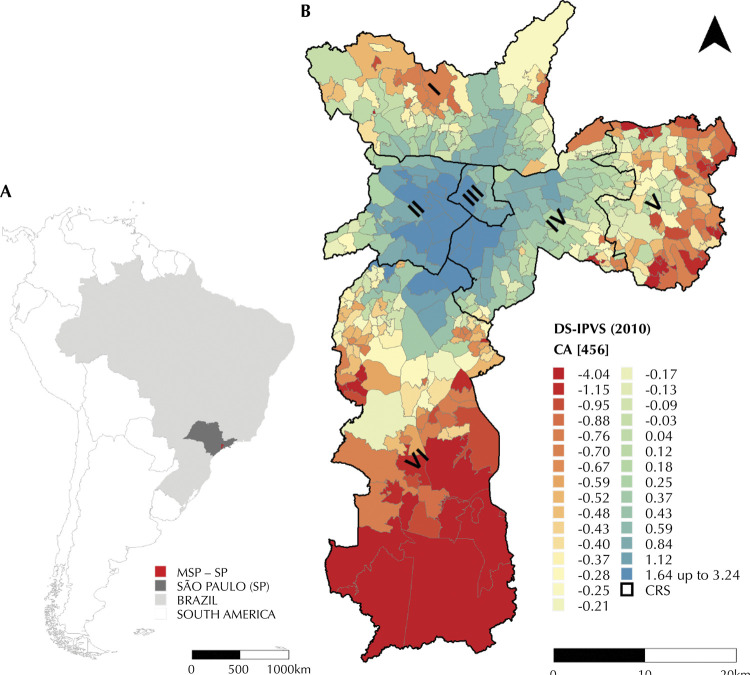
A) Municipality of São Paulo, State of São Paulo, SP, Brazil, South America. B) Distribution of the socioeconomic domain of the São Paulo Social Vulnerability Index (DS-IPVS) according to Areas of Coverage of Basic Health Units. Municipality of São Paulo, 2010.

The study design was ecological, using secondary data, from January 2000 to December 2016. The study area corresponded to the municipality of São Paulo, located in the state of São Paulo (SSP), Brazil (Figure 1A), with population of 11,811,516 inhabitants and population density of 7,765.06 inhabitants/km^2^ in 2019^7^. The units of analysis were the 456 coverage areas of the primary health units in the MSP (CA), version 2015/2016. These areas allow the local health administrator to know the specific conditions of each territory, including sociodemographic, environmental, epidemiological and production characteristics^8^.

The digital mesh of the CA was provided by the Municipal Health Department of São Paulo (SMS-SP). Figure 1B indicates the distribution of the socioeconomic domain (DS-IPVS) for 2010 (demographic census), according to the CA (version 2015/2016). This domain corresponds to the variable factor 1 (v36), referring to the socioeconomic factor of the *Índice Paulista de Vulnerabilidade Social* (IPVS – São Paulo Index of Social Vulnerability), calculated to each census sector (CS). The higher this value, the better the socioeconomic level. The IPVS is an instrument that can be used to evaluate public policies, since it provides greater detail, according to the spatial location, of the living conditions of the municipality, such as vulnerability to poverty^9^.

To estimate the DS-IPVS by CA, a geographic operation was performed, in order to verify the proportion of each CS inserted in each CA. This proportion was applied to the resident population and multiplied by the variable v36. Then, each product belonging to the geographic delimitation of the CA was added, and this value was divided by the sum of the population of that area, thus obtaining a weighted mean. It is noteworthy that 1,537 of the total of 18,923 CS were classified as ignored, since they are areas without population or with reduced population. These CS were disregarded when elaborating the indicator.

Figure 1B also shows the *Coordenadorias Regionais de Saúde* (CRS – Regional Health Coordinators), being: I – North; II – West; III – Downtown; IV – Southeast; V – East; and VI – South.

Information regarding deaths from breast cancer (BC) (CID-10 from C50 to C50.9) and cervical cancer (CC) (CID-10 from C53 to C53.9) of women living in the MSP aged 20 years or over were obtained by formal request to SMS-SP^10^.

The addresses of the deaths were geocoded, using mainly the *Padronizador de Endereços da Prefeitura Municipal de São Paulo* (PAD-PMSP – Address Standardizer of the São Paulo City Hall), by Prodam. This application uses official and unofficial databases, with the ability to process large volumes of records. The addresses not geocoded in the standardizer were geocoded by geolocation platforms that used Google Maps as base for streets and by an application that uses the Navteq street base.

Demographic information was obtained from the Brazilian Institute of Geography and Statistics (IBGE) for the census years from 2000 to 2010, initially aggregated by CS and posteriorly attributed to the CA through a geographical operation between the layers, attributing values proportional to the areas.

Then, we calculated the mortality rates standardized by age, using the direct method and the standard population of the World Health Organization (WHO)^11^, considered the most adequate for developing countries. All referred mortality rates have magnitude “per 100 thousand women aged 20 years or older-year” but were described as “100 thousand women-year. These rates were calculated for the whole period (2000–2016) and also for six periods (2000–2002, 2003–2005, 2006–2008, 2009–2011, 2012–2014 and 2015–2016). These groupings were used to reduce the random fluctuation of data and to perform the visual comparison of the rates according to the units of analysis, arranged in thematic maps.

The maps were also represented by a rate smoothing method – local empirical Bayesian – elaborated from the distance neighborhood matrix, with the five neighbors’ criterion (value corresponding to the average of neighbors), using the expected deaths, standardized by age, as the event of interest in the numerator. The estimate for obtaining the expected deaths was calculated by multiplying the sum of the observed population, in a given CA and study period, by the value of the corresponding standardized rate, divided by 100 thousand. In the denominator, we used the population corresponding to the middle of the period (population of 2010, provided by the census and estimated for each CA). Finally, to obtain annual rates, the smoothed rates were divided by the number of study years.

Age standardization, for the local Bayesian smoothing method, is not usually described in literature; however, considering that age is an important confounding factor in the study of neoplasms, due to age differences between populations, such standardization was necessary.

In addition, we calculated the bivariate local and global Moran indexes, in order to verify the existence of spatial agglomeration in the bivariate analysis between standardized mortality rates and socioeconomic domain. The significance value was calculated by the Monte Carlo hypothesis test, with 999 random replications (considered significant if less than 0.05)^12^.

The applications used to geocode the addresses were: *Padronizador de Endereços da Prefeitura Municipal de São Paulo* (PAD-PMSP), API Google, BatchGeo and Maptitude. Neighborhood matrix, local empirical Bayesian rates and bivariate local and global Moran indexes were calculated in the GeoDa application, version 1.12.1.161. The construction and manipulation of databases, the population estimates for non-census years, the estimates of standardized mortality rates by age and all the elaboration of thematic maps and graphs were performed in the R Studio software, version 1.3.1093. Thematic maps used the “quantile” category, except for the map with the Moran indexes, which was shown as a “categorical” criterion. Geocoding operations were performed in the QGIS software, version 2.18.

This study was linked to a larger project, approved by the Research Ethics Committee of the Faculdade de Saúde Pública of Universidade de São Paulo (CAAE: 76049317.7.0000.5421; opinion number: 2.412.427) and of the City Hall of São Paulo (CAAE: 76049317.7.3001.0086; opinion number: 2.467.549).

## RESULTS

In all, 24,120 address records were geocoded out of a total of 24,386 (success rate of 98.9%). Of these, 19,652 were related to BC (99.0% of 19,856 records) and 4,468 to CC (98.6% of 4,530 records).

**Figure 2:**
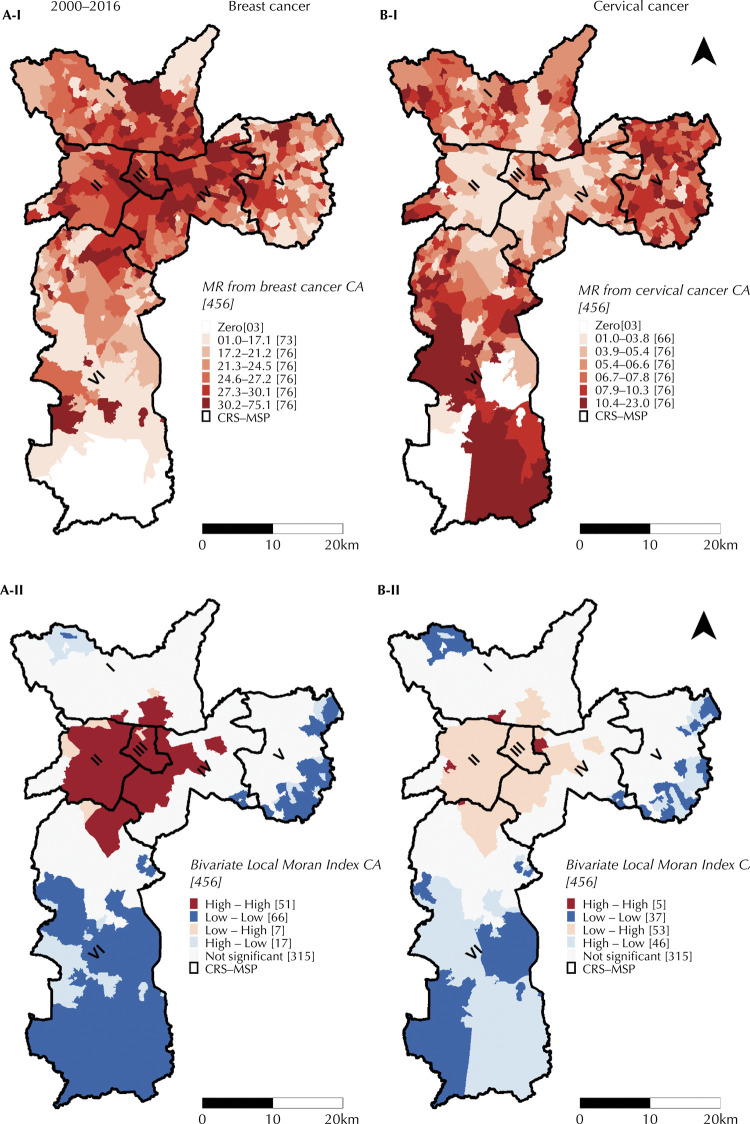
I) Local empirical Bayesian mortality rate (/100,000 women aged 20 years and over – year) standardized by age, according to the year of death. Municipality of São Paulo, 2000–2016 (three years and two years). A-I) for breast cancer. B-I) for cervical cancer. II) Spatial agglomerates of the Bivariate Local Moran Index - mortality rate standardized by age, in relation to the socioeconomic domain of the São Paulo Social Vulnerability Index (DS-IPVS) - according to Areas of Coverage of Basic Health Units. Municipality of São Paulo, 2000–2016. A-II) for breast cancer. B-II) for cervical cancer.

Figures 2A-I and 2B-I show the average local empirical Bayesian mortality rates according to CA, over the entire period. For BC, there is a concentration of higher rates in the West (central part), Downtown and Southeast (upper part) CRS and in a portion of the North CRS. As for CC, the highest rates are concentrated in the South, East and North CRS.

In 2010, the regions of the MSP with the best socioeconomic conditions were the West and Downtown CRS (Figure 1B). A decline in socioeconomic level is perceived the more peripheral the areas are (or towards the periphery). The comparison of these conditions with mean local empirical Bayesian mortality rates, over the entire period, is shown in Figures 2A-II and 2B-II, for BC and CC, respectively.

When comparing Figures 2A-II and 2B-II we observed: (i) a spatially positive and significant relation between mortality from BC and the socioeconomic conditions given by the DS-IPVS, and (ii) a negative and significant relation between mortality from CC and DS-IPVS. Therefore, it is noted that areas with high mortality rates from BC, in general, have neighboring areas with high DS-IPVS (better socioeconomic conditions).

For BC, the areas with a “high-high” pattern (high mortality rates and high DP-IPVS values in the neighbors) were concentrated in the West, Downtown and Southeast CRS. The “low-low” areas (low mortality rate and low DS-IPVS values in the neighbors) were concentrated in the most peripheral regions of the South and East CRS.

For the CC, in the West, Downtown and part of the Southeast CRS, areas of “low-high” pattern predominated (low mortality rates and high DS-IPVS values in the neighbors). In the North (upper), East and South CRS, there was a predominance of “high-low” agglomerates (high mortality rates and low DS-IPVS values in the neighbors), mixed with “low-low” agglomerates (low mortality rates and low DS-IPVS values in the neighbors).

**Figure 3:**
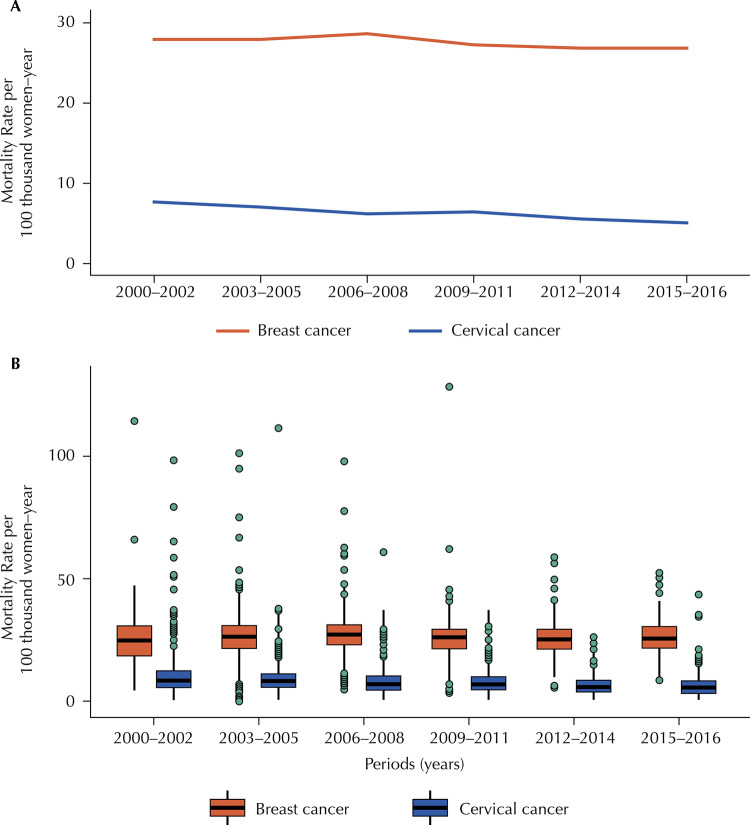
A) Mortality rate (/100 thousand women aged 20 years and over – year) due to breast and cervical cancers, standardized by age, according to the year of death. Municipality of São Paulo, 2000–2016 (three years and two years). B) Local empirical Bayesian mortality rate (/ 100,000 women aged 20 years and over – year) from breast and cervical cancers, standardized by age, second year of death and Areas of Coverage of Basic Health Units. Municipality of São Paulo, 2000–2016 (three years and two years).

Figures 3A and 3B show the temporal evolution of the mortality rates standardized for both cancers, in the MSP as a whole and according to the CA, respectively. There is a trend of increase in mortality from BC until the period 2006-2008, followed by a slight decrease (Figure 3A). As for mortality from CC, there was a noticeable decrease between the beginning and the end of the period, with slight stabilization in 2009–2011.

In Figure 3B, it is possible to identify that, for mortality from BC, there was a trend, over time, of reduced variability of the data, identified by a decrease in the amplitude of outlier values. For mortality from CC, variation was higher in the first period (2000-2002) than in the others, with a gradual, but discrete reduction of the median value over time. In both cancers, the distance between the first and third quartiles narrowed over time.

**Figure 4:**
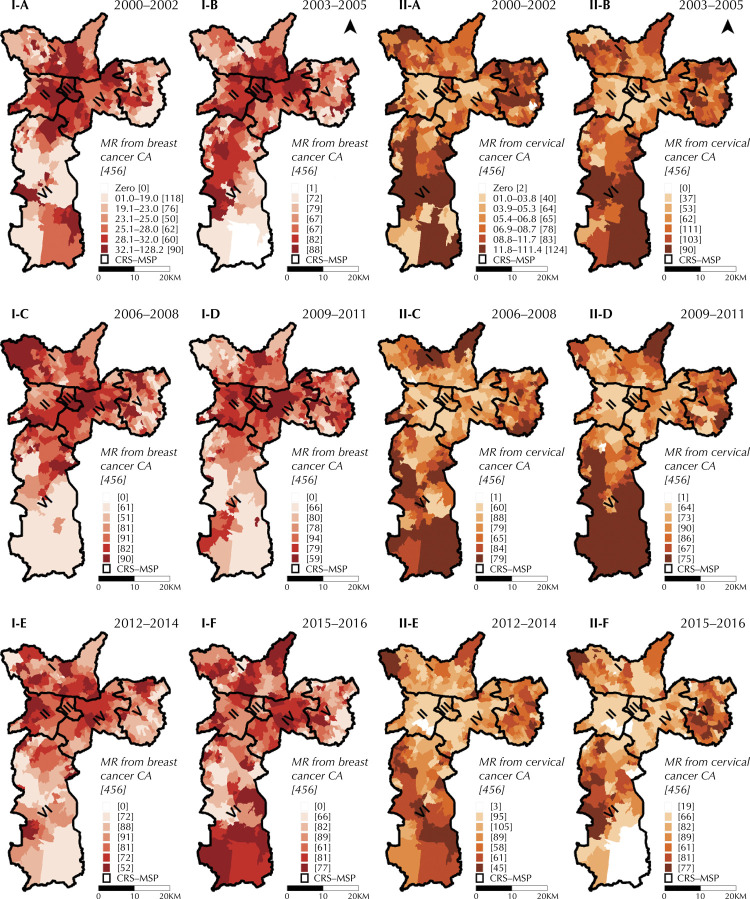
Mortality rates (MR) (/100 thousand women aged 20 years and over – year) standardized by age, according to year of death and Areas of Coverage of Basic Health Units. Municipality of São Paulo, 2000–2016 (three years and two years). I-A) For breast cancer 2000–2002. I-B) For breast cancer 2003–2005. I-C) For breast cancer, 2006-2008. I-D) For breast cancer, 2009–2011. I-E) For breast cancer, 2012–2014.I-F) For breast cancer, 2015–2016. II-A) For cervical cancer, 2000–2002. II-B) For cervical cancer, 2003–2005. II-C) For cervical cancer, 2006–2008. II-D) For cervical cancer, 2009–2011. II-E) For cervical cancer, 2012–2014. II-F) For cervical cancer, 2015–2016.

Temporal evolution of mortality rates standardized for BC and CC is also shown as in the form of maps according to CA (Figure 4). We observed, over time, that the pattern of spatial distribution of mortality due to BC was inconstant. In the Downtown and West CRS, an apparent reduction in mortality was observed over the years. In addition, the number of areas with rates above 32 deaths per 100 thousand women-year, last category, represented in the darkest color on the maps, decreased over time. This pattern is coherent with the curve of the entire MSP, shown in Figure 3A; however, it is worth noting that the last period only comprises two years.

In general, over time, the pattern of spatial distribution of mortality from CC remained with the highest mortality rates located in peripheral regions. However, but also coherent with Figure 3A, there is a tendency of reduced mortality rate from this cancer, in terms of geographical and value dispersion, over the years of the study. The large number of areas with value 0 in the last period (2015–2016) stands out, with 19 CA without record of deaths from CC.

The Table shows the overall values, for both cancers, of mortality rates standardized by age; rates stratified by age groups, with a ratio of comparison between them; and mortality associated to socioeconomic conditions (DS-IPVS). It is noteworthy that mortality values for BC were always higher, with an increase in this difference as the age stratum increases. In addition, the global Moran index reaffirms the results of the direct association of DS-IPVS with mortality from BC, and inverse with that from CC.

**Table:** Distribution of overall mortality rates, ratio between rates and bivariate global Moran index, according to breast and cervical cancer. Municipality of São Paulo, 2000–2016.

Cancer mortality	Breast cancer (BC)	Cervical cancer (CC)	BC/CC ratio
Overall rates (deaths per 100 thousand women-year)			
Overall standardized rate 20 years or older	27.6	6.3	4.4
Overall rate 20 to 39 years	3.6	1.6	2.3
Overall rate 40 to 69 years	37.6	9.1	4.1
Overall rate 70 years or older	113.0	18.3	6.2
Association with the socioeconomic conditions of the neighborhood			
Bivariate global Moran index	0.34 (p < 0.01)	- 0.34 (p < 0.01)	

In the year-by-year analysis, we found that the younger age group showed a trend of stability in mortality from BC, but of increased mortality from CC, especially after the period 2006-2008. On the other hand, the age group from 40 to 69 years showed a tendency of stability for mortality from BC and of decline for mortality from CC. In the age group of 70 years of over, mortality from BC denoted a slight downward trend, with peaks in the periods 2006–2008 and 2015–2016, while mortality from CC showed a sharp downward trend, peaking in the period 2009–2011.

## DISCUSSION

This study allowed to evidence the spatial pattern of mortality from breast and cervical cancer over time, associated with socioeconomic conditions of the MSP. With this, we identified that BC, throughout the period, denotes a pattern, with higher rates located in the central regions with better socioeconomic conditions, but with a decrease at the end of the period and apparent change of pattern, and increased mortality in peripheral regions. On the other hand, the highest rates of mortality from CC continued in peripheral regions with worse socioeconomic conditions, despite decreasing over the study period.

Therefore, an important finding of this study is that, for BC, in the entire period, there was a direct relation between areas with higher mortality rates and areas with better socioeconomic conditions. For CC, the behavior was the opposite. These relations were expressed not only descriptively, but also with the significant values of the global and local Moran indexes.

This result agrees with the descriptive study performed by Neves and Naffah^6^, which evidenced an apparent association between areas with high mortality from BC and areas with better socioeconomic conditions, and the inverse pattern for CC, in the MSP, from 2010–2012. Another ecological study, conducted by Rocha-Brischiliari et al.^13^, found, in the state of Paraná, in 2009–2012, a positive and significant relation between mortality from BC and greater access to health services; and a negative one in relation to the illiteracy rate. Similarly, in the state of São Paulo, between 2006–2012, Diniz et al.^14^ found a positive and significant association between areas with high mortality from BC and areas with high mammography rates, higher nulliparity and greater private medical care.

The causes of increased mortality for women with better socioeconomic situation may be linked to the higher exposure to carcinogens, and lower occurrence of protective factors in this group^14^. As they have more access to health services, they are more exposed to factors associated with breast cancer, such as hormonal contraception, hormone replacement therapy in menopause and a higher concentration of mammograms (increased ionizing radiation) and breast biopsies. Because they are more subject to overdiagnosis, they may suffer more from the cardiotoxic effects of chemotherapy and hormone therapy, as well as the carcinogenic effects of radiotherapy^15^.

Studies allow to estimate that, when adding damages potentially associated with cancer treatments, despite the decreased mortality due to the specific cause (breast cancer), there may be an increase in mortality due to the set of causes, mainly due to increased cardiovascular mortality and from other cancers^16^. In addition, the reproductive characteristics of these women are less protective: they have fewer children (therefore, with lower chances of breastfeeding) and have them later in life^17^. In the same line, a meta-analysis and systematic review study conducted in Europe pointed to a higher and significant mortality from BC in women with higher socioeconomic level. As explanation, they listed reproductive factors, mammographic screening, hormone replacement therapy and lifestyle-related factors^18^.

Our study also showed that, for BC, there was a downward trend in the rates of the municipality at the end of the analyzed period. In addition, over time, there was a narrowing of data variability per unit of analysis. Also, we observed an apparent change in the spatial distribution of mortality, with increased rates in peripheral regions and reduction in central regions of the MSP. Thus, analyzing all these results together, we expect that, currently, the spatial pattern of mortality from BC tends to a uniform distribution in the municipality.

Similarly, mortality from BC has been declining in developed countries^19-21^, which is associated with less aggressive, more effective and safe treatments and a reduced use of estrogen replacement therapy in these populations^9,22,23^. It has also been declining in Brazil and in the Southeast region of the country, despite increasing trends in the North and Northeast regions^24,25^. Barbosa et al.^24^ indicated, in their study on mortality projections for this cancer until 2030, that the highest rates will be recorded in the less developed regions of Brazil. Late diagnosis is placed, by that and another study^24,26^, as a possible explanation for the increased mortality expected in poorer regions. However, there may also be an association between greater coverage of mammographic screening and increased mortality from BC (due to aspects such as overdiagnosis and over-treatment^14,16,22,23^).

The ecological study of Harding et al.^22^ in the United States, comparing the regions and the screening rates among more than 16 million women, found that, the higher the screening rate by mammography, the higher the incidence of breast cancer, without implying a reduction in mortality from this cancer. That study also found a non-significant association between screening and increased mortality specifically from breast cancer. In the study by Rocha-Brischiliari et al.^13^, not only was there an association between the greater use of diagnostic services and higher mortality, but they also noted an increased mortality when there is greater proximity to specialized services in the patients’ housing region. Those findings go in the same direction as in our study and may help explain why increased screening coverage is associated with an increased mortality in poorer areas.

For mortality from CC, there is also a decrease of values over time for the entire MSP, and apparent reduction in dispersion in the units of analysis, but without changes in the spatial pattern. That is, the highest rates continue to be in the more peripheral regions throughout the study period. This is an equally important result; the decrease is probably explained by the increased screening coverage in the municipality, which provides detection and treatment of precursor lesions, although it is still unequal, with less access in peripheral regions^27,18^. This decrease agrees with the temporal trend profile of mortality from this cancer in Brazil and its regions, except for the North region^25,29^. In relation to the analyses stratified by age group, the youngest group (20–39 years) was the only with an increase trend, a phenomenon that has also been observed in European countries^30^.

It is also worth considering that the value shown regarding the overall mortality rate for BC in the municipality was more than twice the world average in 2018. As for CC, the value found was slightly lower than the world average. It is noteworthy that these world rates are standardized according to the same standard population, but without the filter for women aged 20 years or over^1^.

Some limitations of this study are related to the use of secondary data, subject to different degrees of information quality; and to the estimates made to calculate the population of non-census years, since the precision of these estimates decreases as they distance themselves from census years. The following are also limitations: (i) non-consideration of census sectors with less than 50 permanent private households to calculate the IPVS of the CA, and (ii) extrapolation of information from the census sector level to these units of analysis, since the census delimitations do not correspond to those of the CA. This problem was solved with spatial analysis tools. In addition, there is the issue of random fluctuation, which occurs due to the existence of areas with small populations, minimized by the use of local empirical Bayesian methodology and grouping into periods.

As strengths, we highlight that the estimates relied on geoprocessing tools, and that a wide study period was used. In addition, the quality of mortality records in the MSP resulted in a high success rate of geocoding (98.9%), indicating, among other factors, a good completion of death certificates.

Knowledge on spatial and temporal patterns allows a better targeting of resources to prevent and promote health in the territories. It also raises questions that may be more explored by other studies aimed at better clarifying the reasons for the different behaviors.
