# Supplementary material for: Spatial pattern of mortality from breast and cervical cancer in the city of São Paulo
Source: Rev Saude Publica. 2020 Dec 4;54:142. doi: 10.11606/s1518-8787.2020054002447 (PMC7726920; doi:10.11606/s1518-8787.2020054002447)
Supplement: Translation

# Translation: Padrão espacial da mortalidade por câncer de mama e colo do útero na cidade de São Paulo

**Authors:** Patricia Marques Moralejo Bermudi, Alessandra Cristina Guedes Pellini, Elizabeth Angélica Salinas Rebolledo, Carmen Simone Grilo Diniz, Breno Souza de Aguiar, Adeylson Guimarães Ribeiro, Marcelo Antunes Failla, Oswaldo Santos Baquero, Francisco Chiaravalloti

**Affiliations:** I Brasil Universidade de São Paulo. Faculdade de Saúde Pública. Departamento de Epidemiologia. São Paulo, SP, Brasil; II Brasil Universidade Nove de Julho. Faculdade de Medicina. Diretoria de Ciências Médicas. São Paulo, SP, Brasil; III Brasil Universidade de São Paulo. Faculdade de Saúde Pública. Programa de Pós-Graduação em Saúde Pública. São Paulo, SP, Brasil; IV Brasil Universidade de São Paulo. Faculdade de Saúde Pública. Departamento de Saúde, Ciclos de Vida e Sociedade. São Paulo, SP, Brasil; V Brasil Secretaria Municipal de Saúde de São Paulo. Coordenação de Epidemiologia e Informação. Gerência de Geoprocessamento e Informações Socioambientais. São Paulo, SP, Brasil; VI Brasil Hospital de Câncer de Barretos. Instituto de Ensino e Pesquisa. Barretos, SP, Brasil; VII Brasil Universidade de São Paulo. Faculdade de Medicina Veterinária e Zootecnia. Departamento de Medicina Veterinária Preventiva e Saúde Animal. São Paulo, SP, Brasil

**Keywords:** Neoplasias da Mama, Neoplasias do Colo do Útero, Análise Espacial, Análise Socioeconômica, Mortalidade

## Abstract

**OBJETIVOS:**

Verificar o padrão espacial da mortalidade pelos cânceres de mama e do colo do útero, em áreas da atenção primária à saúde, levando em consideração as condições socioeconômicas.

**MÉTODOS:**

O estudo é ecológico, de janeiro de 2000 a dezembro de 2016. A área de estudo é o município de São Paulo, Brasil, e suas 456 áreas de abrangência das unidades básicas de saúde. As informações sobre óbitos de mulheres com 20 anos ou mais de idade foram geocodificadas segundo endereço de residência. Foram calculadas as taxas de mortalidade, padronizadas por idade, e suavizadas pelo método bayesiano empírico local, além de agrupadas em três ou dois anos para reduzir a flutuação aleatória dos dados. Além disso, foram calculados os índices de Moran global e local bivariados, para verificar a existência de aglomeração espacial das taxas de mortalidade padronizadas com um domínio de condição socioeconômica, elaborado a partir do Índice Paulista de Vulnerabilidade Social.

**RESULTADOS:**

A taxa de sucesso da geocodificação foi de 98,9%. A mortalidade por câncer de mama, sem estratificação por tempo, apresentou um padrão com maiores taxas localizadas nas regiões centrais e com melhores condições socioeconômicas. Apresentou queda ao final do período e mudança de padrão espacial, com aumento da mortalidade nas regiões periféricas. Já a mortalidade por câncer do colo do útero manteve-se com as maiores taxas nas regiões periféricas e com piores condições socioeconômicas, apesar de apresentar redução ao longo do tempo.

**CONCLUSÃO:**

O padrão espacial da mortalidade pelos cânceres do estudo, ao longo do tempo, sugere associação com as melhores condições socioeconômicas do município, seja como proteção (colo) ou risco (mama). Esse conhecimento pode direcionar recursos para a prevenção e a promoção da saúde nos territórios.

## INTRODUÇÃO

Os cânceres de mama e do colo do útero são importantes problemas mundiais de saúde pública. No Brasil, o câncer de mama na população feminina apresentou aumento entre as estimativas de 2018–2019 e de 2020–2022, e se manteve como o mais incidente, à exceção do câncer de pele não melanoma. Já o câncer do colo do útero subiu uma posição, e agora é o terceiro mais incidente. Ambos os cânceres também são importantes causas de mortalidade no país^1^.

No município de São Paulo (MSP), entre 2008 e 2012 a taxa de mortalidade média anual por câncer de mama, padronizada por idade, foi de 16,2 por 100 mil mulheres-ano, e para o câncer do colo do útero, de 3,7 óbitos por 100 mil mulheres-ano, com tendência de queda da mortalidade do primeiro câncer e estabilidade do segundo^4^.

Alguns estudos realizados no MSP, nos períodos de 1985–1999 e 2010–2012, verificaram que regiões com melhores condições socioeconômicas apresentaram risco mais elevado de mortalidade por câncer da mama. De forma oposta, regiões com piores condições indicaram risco mais elevado de mortalidade por câncer do colo do útero quando comparadas aos demais territórios analisados^5,6^.

Este estudo tem como objetivo verificar o padrão espacial da mortalidade pelos cânceres de mama e do colo do útero em mulheres com 20 anos ou mais de idade, residentes no MSP, no período de 2000 a 2016, realizando a comparação entre esses cânceres e levando em consideração as condições socioeconômicas do município, em áreas de abrangência da atenção primária à saúde.

## MÉTODOS

O delineamento do estudo foi ecológico, com uso de dados secundários, no período de janeiro de 2000 a dezembro de 2016. A área de estudo correspondeu ao município de São Paulo, localizado no estado de São Paulo (ESP), Brasil (Figura 1A), com população de 11.811.516 habitantes e densidade demográfica de 7.765,06 habitantes/km^2^ em 2019^7^. As unidades de análise foram as 456 áreas de abrangência das unidades básicas de saúde do MSP (AA), versão 2015/2016. Essas áreas permitem ao gestor de saúde local conhecer as condições específicas de cada território, incluindo características sociodemográficas, ambientais, epidemiológicas e de produção^8^.

**Figura 1:** A) Município de São Paulo, Estado de São Paulo - SP, Brasil, América do Sul. B) Distribuição do domínio socioeconômico do Índice Paulista de Vulnerabilidade Social (DS-IPVS) segundo Áreas de Abrangência das Unidades Básicas de Saúde. Município de São Paulo, 2010.

A malha digital das AA foi fornecida pela Secretaria Municipal de Saúde de São Paulo (SMS-SP). A Figura 1B indica a distribuição do domínio socioeconômico (DS-IPVS) do ano de 2010 (censo demográfico), segundo as AA (versão 2015/2016). Este domínio corresponde à variável fator 1 (v36), referente ao fator socioeconômico do Índice Paulista de Vulnerabilidade Social (IPVS), calculado para cada setor censitário (SC). Quanto maior for este valor, melhor é o nível socioeconômico. O IPVS é instrumento que pode ser usado para avaliar políticas públicas, uma vez que permite maior detalhamento, segundo a localização espacial, das condições de vida do município, como vulnerabilidade a pobreza^9^.

Para estimar o DS-IPVS por AA, foi realizada operação geográfica, de modo a se verificar a proporção de cada SC inserida em cada AA. Esta proporção foi aplicada à população residente e multiplicada pela variável v36. Em seguida, foi somado cada produto pertencente à delimitação geográfica das AA, e este valor foi dividido pela soma da população daquela área, obtendo-se, assim, uma média ponderada. Destaca-se que 1.537 do total de 18.923 SC foram classificados como ignorados, pois são áreas sem população ou com população reduzida. Esses SC foram desconsiderados para a elaboração do indicador.

Na Figura 1B exibem-se também as Coordenadorias Regionais de Saúde (CRS), sendo: I – Norte; II – Oeste; III – Centro, IV – Sudeste; V – Leste; e VI – Sul.

As informações referentes a óbitos por câncer de mama (CM) (CID-10 de C50 a C50.9) e câncer do colo do útero (CCU) (CID-10 de C53 a C53.9) de mulheres residentes no MSP com 20 anos ou mais de idade foram obtidas por meio de solicitação formal à SMS-SP^10^.

Os endereços dos óbitos foram geocodificados utilizando principalmente o Padronizador de Endereços da Prefeitura Municipal de São Paulo (PAD-PMSP), da Prodam. Este aplicativo utiliza bases de dados oficiais e não oficiais, com capacidade para processar grandes volumes de registros. Os endereços não geocodificados no padronizador foram geocodificados por plataformas de geolocalização que utilizam o Google Maps como base de logradouros e por aplicativo que dispõe da base de logradouros Navteq.

As informações demográficas foram obtidas do Instituto Brasileiro de Geografia e Estatística (IBGE) para os anos censitários de 2000 e 2010, inicialmente agregadas por SC e posteriormente atribuídas às AA por meio de uma operação geográfica entre as camadas, atribuindo-se valores proporcionais às áreas.

A seguir, foram calculadas as taxas de mortalidade padronizadas por idade, utilizando o método direto e a população padrão da Organização Mundial da Saúde (OMS)^11^, considerada a mais adequada para países em desenvolvimento. Todas as taxas de mortalidade referidas têm a grandeza “por 100 mil mulheres com 20 anos ou mais de idade-ano”, mas foram descritas como “100 mil mulheres-ano”. Essas taxas foram calculadas para o período todo (2000–2016) e também para seis períodos (2000–2002, 2003–2005, 2006–2008, 2009–2011, 2012–2014 e 2015–2016). Utilizaram-se esses agrupamentos para reduzir a flutuação aleatória dos dados e realizar a comparação visual das taxas segundo as unidades de análise, dispostas em mapas temáticos.

Os mapas foram representados também por um método de suavização de taxas – Bayesiano empírico local –, elaborado a partir da matriz de vizinhança de distância, com o critério de cinco vizinhos (valor correspondente à média de vizinhos), utilizando-se os óbitos esperados, padronizados por idade, como o evento de interesse no numerador. A estimativa para a obtenção dos óbitos esperados foi calculada pela multiplicação da somatória da população observada, em determinada AA e período de estudo, pelo valor da taxa padronizada correspondente, dividida por 100 mil. Já no denominador foi utilizada a população correspondente ao meio do período (população de 2010, fornecida pelo censo e estimada para cada AA). Ao final, para se obterem as taxas anuais, as taxas suavizadas foram divididas pelo número de anos do estudo.

A padronização por idade, para o método de suavização Bayesiano local, não é comumente descrita na literatura; contudo, tendo em vista que a idade é importante fator de confusão no estudo de neoplasias, em função das diferenças etárias entre as populações, tal padronização foi necessária.

Além disso, foram calculados os índices de Moran global e local bivariados, para verificar a existência de aglomeração espacial na análise bivariada entre as taxas de mortalidade padronizadas e o domínio socioeconômico. O valor da significância foi calculado pelo teste de hipóteses de Monte Carlo, com 999 replicações aleatórias (considerado significativo se inferior a 0,05)^12^.

Os aplicativos utilizados para a geocodificação dos endereços foram: Padronizador de Endereços da Prefeitura Municipal de São Paulo (PAD-PMSP), API Google, BatchGeo e Maptitude. A matriz de vizinhança, as taxas Bayesianas empíricas locais e os índices de Moran local e global bivariados foram calculados no aplicativo GeoDa, versão 1.12.1.161. A construção e a manipulação dos bancos de dados, as estimativas populacionais para os anos não censitários, os cálculos das taxas padronizadas de mortalidade por idade e todas as elaborações de mapas temáticos e gráficos foram realizados no *software* R Studio versão 1.3.1093. Os mapas temáticos utilizaram a categoria de “quantil”, exceto o mapa com os índices de Moran, que foi apresentado com o critério “categórico”. As operações de geocodificação foram feitas no *software* QGIS versão 2.18.

Este estudo foi vinculado a um projeto maior, aprovado pelos Comitês de Ética da Faculdade de Saúde Pública da Universidade de São Paulo (CAAE: 76049317.7.0000.5421; número do parecer: 2.412.427) e da Prefeitura de São Paulo (CAAE: 76049317.7.3001.0086; número do parecer: 2.467.549).

## RESULTADOS

Ao todo, foram geocodificados 24.120 registros de endereços do total de 24.386 (taxa de sucesso de 98,9%). Destes, 19.652 foram referentes ao CM (99,0% de 19.856 registros) e 4.468 ao CCU (98,6% de 4.530 registros).

As Figuras 2A-I e 2B-I apresentam as taxas médias de mortalidade Bayesianas empíricas locais segundo AA, em todo o período. Para o CM, nota-se uma concentração de taxas maiores nas CRS Oeste (parte mais central), Centro e Sudeste (parte superior) e em uma parcela da CRS Norte. Já para o CCU, as maiores taxas estão concentradas nas CRS Sul, Leste e Norte.

**Figura 2:** I) Taxas de mortalidade Bayesiana empírica local (/ 100.000 mulheres com 20 anos ou mais - ano) padronizadas por idade, de acordo com o ano do óbito. Município de São Paulo, 2000–2016 (três anos e dois anos). A-I) para câncer de mama. B-I) para câncer do colo do útero. II) Aglomerados espaciais do Índice de Moran Local Bivariado - taxas de mortalidade padronizadas por idade, em relação ao Domínio Socioeconômico do Índice Paulista de Vulnerabilidade Social (DS-IPVS), segundo Áreas de Abrangências das Unidades Básicas de Saúde. Município de São Paulo, 2000–2016. A-II) para câncer de mama. B-II) para câncer do colo do útero.

No ano de 2010, as regiões do MSP com melhores condições socioeconômicas foram as CRS Oeste e Centro (Figura 1B). Percebe-se um declínio do nível socioeconômico quanto mais periféricas são as áreas (ou em direção à periferia). A comparação dessas condições com as taxas médias de mortalidade Bayesianas empíricas locais, no período todo, está indicada nas Figuras 2A-II e 2B-II, para CM e CCU, respectivamente.

Ao se compararem as 2A-II and 2B-II, observam-se: (i) uma relação espacialmente positiva e significante entre a mortalidade por CM e as condições socioeconômicas dadas pelo DS-IPVS e (ii) uma relação negativa e significante entre a mortalidade por CCU e o DS-IPVS. Logo, nota-se que áreas com elevadas taxas de mortalidade por CM, em geral, possuem áreas vizinhas com DS-IPVS elevado (melhores condições socioeconômicas).

Para o CM, as áreas com padrão “alto-alto” (taxas de mortalidade altas e valores altos de DS-IPVS nos vizinhos) concentraram-se nas CRS Oeste, Centro e Sudeste. Já as áreas “baixo-baixo” (taxas de mortalidade baixas e valores baixos de DS-IPVS nos vizinhos) concentraram-se nas regiões mais periféricas das CRS Sul e Leste.

Para o CCU, nas CRS Oeste, Centro e parte da Sudeste, predominaram áreas de padrão “baixo-alto” (taxas de mortalidade baixas e valores altos de DS-IPVS nos vizinhos). Nas CRS Norte (superior), Leste e Sul, houve predominância de aglomerados “alto-baixo” (taxas de mortalidade altas e valores baixos de DS-IPVS nos vizinhos), mesclados com aglomerados “baixo-baixo” (taxas de mortalidade baixas e baixos valores de DS-IPVS nos vizinhos).

As Figuras 3A e 3B mostram a evolução temporal das taxas de mortalidade padronizadas para ambos os cânceres, no MSP como um todo e segundo as AA, respectivamente. Denota-se tendência de aumento da mortalidade por CM até o período 2006–2008, seguida por leve queda (Figura 3A). Quanto à mortalidade por CCU, houve visível queda entre o início e o fim do período, com leve estabilização em 2009–2011.

**Figura 3:** A) Taxas de mortalidade (/100 mil mulheres com 20 anos ou mais de idade - ano) por cânceres de mama e do colo do útero, padronizadas por idade, segundo ano do óbito. Município de São Paulo, 2000- 2016 (triênios e biênio). B) Taxa de mortalidade Bayesiana empírica local (/100 mil mulheres com 20 anos ou mais de idade - ano) por cânceres de mama e do colo do útero, padronizada por idade, segundo ano do óbito e Áreas de Abrangência das Unidades Básicas de Saúde. Município de São Paulo, 2000-2016 (triênios e biênio).

Na Figura 3B, é possível identificar que, para a mortalidade por CM, ocorreu tendência, ao longo do tempo, de redução da variabilidade dos dados, identificada por queda da amplitude dos valores *outliers*. Para a mortalidade por CCU, a variação foi mais elevada no primeiro período (2000–2002) do que nos demais, e houve redução gradual, mas discreta, do valor da mediana ao longo do tempo. Em ambos os cânceres, ocorreu estreitamento da distância entre o primeiro e o terceiro quartis ao longo do tempo.

A evolução temporal das taxas de mortalidade padronizadas por CM e CCU também é apresentada na forma de mapas segundo AA (Figura 4). Observa-se, ao longo do tempo, que o padrão de distribuição espacial da mortalidade por CM não foi constante. Nas CRS Centro e Oeste verificou-se aparente redução da mortalidade ao longo dos anos. Além disso, a quantidade de áreas com taxas acima de 32 óbitos por 100 mil mulheres-ano, última categoria, representada na cor mais escura nos mapas, diminuiu com o tempo. Este padrão é coerente com a curva de todo o MSP, apresentada na Figura 3A; no entanto, vale ressaltar que o último período compreende somente dois anos.

**Figura 4:** Taxas de mortalidade (TM) (/100 mil mulheres com 20 anos ou mais de idade - ano) padronizadas por idade, segundo ano do óbito e Áreas de Abrangência das Unidades Básicas de Saúde. Município de São Paulo, 2000-2016 (triênios e biênio). I-A) Por câncer da mama 2000-2002. I-B) Por câncer de mama 2003-2005. I-C) Por câncer de mama, 2006-2008. I-D) Por câncer de mama, 2009-2011. I-E) Por câncer de mama, 2012-2014. I-F) Por câncer de mama, 2015-2016. II-A) Por câncer do colo do útero, 2000-2002. II-B) Por câncer do colo do útero, 2003-2005. II-C) Por câncer do colo do útero, 2006-2008. II-D) Por câncer do colo do útero, 2009-2011. II-E) Por câncer do colo do útero, 2012-2014. II-F) Por câncer do colo do útero, 2015-2016.

De modo geral, ao longo do tempo, o padrão de distribuição espacial da mortalidade por CCU permaneceu com as maiores taxas de mortalidade localizadas nas regiões periféricas. No entanto, mas também coerente com a Figura 3A, nota-se uma tendência de redução da taxa de mortalidade por esse câncer, em termos de dispersão geográfica e de valores, ao longo dos anos de estudo. Destaca-se a grande quantidade de áreas com valor 0 no último período (2015–2016), com 19 AA sem registro de mortes por CCU.

A Tabela apresenta os valores globais, para ambos os cânceres, das taxas de mortalidade padronizadas por idade; taxas estratificadas por faixas etárias, com uma razão de comparação entre estas; e das mortalidades associadas a condições socioeconômicas (DS-IPVS). Chama atenção que os valores de mortalidade para o CM foram sempre maiores, com um incremento dessa diferença conforme aumenta o estrato etário. Além disso, o índice de Moran global reafirma os resultados da associação direta do DS-IPVS com a mortalidade por CM, e inversa com aquela por CCU.

**Tabela:** Distribuição das taxas globais de mortalidade, da razão entre taxas e do índice de Moran global bivariado, segundo os tipos de câncer, de mama e do colo do útero. Município de São Paulo, 2000–2016.

Mortalidade por câncer	Mama (CM)	Colo do útero (CCU)	Razão CM/CCU
Taxas globais (óbitos a cada 100 mil mulheres-ano)			
Taxa padronizada global 20 anos e mais de idade	27,6	6,3	4,4
Taxa global 20 a 39 anos de idade	3,6	1,6	2,3
Taxa global 40 a 69 anos de idade	37,6	9,1	4,1
Taxa global 70 anos e mais de idade	113,0	18,3	6,2
Associação com as condições socioeconômicas da vizinhança			
Índice de Moran global bivariado	0,34 (p < 0,01)	- 0,34 (p < 0,01)	

Na análise ano a ano, constatou-se que a faixa etária mais jovem apresentou tendência de estabilidade na mortalidade por CM, porém de aumento da mortalidade por CCU, principalmente após o período 2006–2008. Já a faixa etária de 40 a 69 anos apresentou tendência de estabilidade para a mortalidade por CM e de declínio para a mortalidade por CCU. Na faixa etária de 70 anos ou mais, a mortalidade por CM denotou leve tendência de queda, com picos nos períodos de 2006–2008 e 2015–2016, enquanto a mortalidade por CCU apresentou acentuada tendência de queda, com pico no período 2009–2011.

## DISCUSSÃO

Este estudo possibilitou evidenciar o padrão espacial da mortalidade pelos cânceres de mama e do colo do útero ao longo do tempo, em associação com as condições socioeconômicas do MSP. Com isso, identificou-se que o CM, em todo o período, denota um padrão, com maiores taxas localizadas nas regiões centrais e com melhores condições socioeconômicas, mas com queda ao final do período e aparente mudança de padrão, com aumento da mortalidade nas regiões periféricas. Já a mortalidade por CCU se manteve com as maiores taxas nas regiões periféricas e com piores condições socioeconômicas, apesar de apresentar declínio ao longo do período do estudo.

Portanto, um importante achado deste estudo é que, para o CM, no período todo, houve relação direta entre as áreas com maiores taxas de mortalidade e áreas de melhores condições socioeconômicas. Já para o CCU, o comportamento foi inverso. Essas relações foram expressas não somente de forma descritiva, mas também com os valores significativos dos índices de Moran global e local.

Esse resultado concorda com o estudo descritivo realizado por Neves e Naffah^6^, que evidenciou aparente associação entre áreas com elevada mortalidade por CM e áreas com melhores condições socioeconômicas, e padrão inverso para o CCU, no MSP, no período 2010–2012. Outro estudo ecológico, realizado por Rocha-Brischiliari e colaboradores^13^, encontrou, no estado do Paraná, em 2009–2012, relação positiva e significante entre mortalidade por CM e maior acesso a serviços de saúde; e negativa em relação à taxa de analfabetismo. De maneira semelhante, no estado de São Paulo, entre 2006–2012, Diniz e colaboradores^14^ encontraram associação positiva e significante entre áreas de alta mortalidade por CM e áreas com elevadas taxas de mamografia, maior nuliparidade e maior assistência médica privada.

As causas da mortalidade aumentada para mulheres com melhor situação socioeconômica estão provavelmente ligadas à maior exposição a carcinógenos, e menor ocorrência de fatores de proteção neste grupo^14^. Como têm mais acesso a serviços de saúde, estão mais expostas a fatores associados ao câncer de mama, tais como contracepção hormonal, terapia de reposição hormonal na menopausa e maior concentração de mamografias (aumento da radiação ionizante) e de biópsias mamárias. Por serem mais sujeitas ao sobrediagnóstico, podem sofrer mais os efeitos cardiotóxicos da quimioterapia e da hormonioterapia, assim como os efeitos carcinógenos da radioterapia^15^.

Estudos permitem estimar que, quando adicionados os danos potencialmente associados a tratamentos de câncer, apesar da diminuição da mortalidade pela causa específica (câncer de mama), pode haver aumento da mortalidade pelo conjunto de causas, principalmente em função do aumento da mortalidade cardiovascular e por outros cânceres^16^. Além disso, as características reprodutivas dessas mulheres são menos protetoras: têm menos filhos (portanto, menores chances de amamentação) e os têm mais tardiamente^17^. Na mesma linha, um estudo de metanálise e revisão sistemática realizado na Europa apontou para mortalidade por CM maior e significativa em mulheres com maior nível socioeconômico. Como explicação, foram listados fatores reprodutivos, rastreamento mamográfico, terapia de reposição hormonal e fatores relacionados ao estilo de vida^18^.

O presente estudo também mostrou que, para o CM, ocorreu tendência de queda das taxas do município ao final do período analisado. Além disso, ao longo do tempo, ocorreu estreitamento da variabilidade dos dados por unidade de análise. Ainda, observou-se aparente mudança na distribuição espacial da mortalidade, com aumento das taxas nas regiões periféricas e redução nas regiões centrais do MSP. Assim, analisando todos esses resultados em conjunto, pode-se esperar que, atualmente, o padrão espacial da mortalidade por CM esteja tendendo a uma distribuição uniforme no município.

De forma semelhante, a mortalidade por CM vem caindo em países desenvolvidos^19^, o que está associado a tratamentos menos agressivos, mais efetivos e seguros e a uma redução do uso de terapia de reposição de estrogênio nessas populações^9,22,23^. Também vem caindo no Brasil e na região Sudeste do país, apesar das tendências de aumento nas regiões Norte e Nordeste^24,25^. Barbosa e colaboradores^24^ indicaram, em seu estudo sobre as projeções de mortalidade por esse câncer até 2030, que as maiores taxas serão registradas nas regiões menos desenvolvidas do Brasil. O diagnóstico tardio é colocado, por este e por um outro estudo^24,26^, como possível explicação para o aumento da mortalidade esperada em regiões mais pobres. No entanto, também pode haver associação entre maior cobertura do rastreamento mamográfico e aumento da mortalidade por CM (devido a aspectos como sobrediagnóstico e sobretratamento^14,16,22,23^).

O estudo ecológico de Harding e colaboradores^22^ nos Estados Unidos, comparando as regiões e as taxas de rastreamento entre mais de 16 milhões de mulheres, encontrou que, quanto maior é a taxa de rastreamento por mamografia, maior é a incidência de câncer de mama, sem implicar redução da mortalidade por este câncer. Esse estudo mostrou, ainda, associação não significativa entre rastreamento e aumento da mortalidade específica por câncer de mama. No estudo de Rocha-Brischiliari et al.^13^, não apenas houve associação entre maior uso de serviços de diagnósticos com maior mortalidade, mas também se notou aumento da mortalidade quando há maior proximidade de serviços especializados na região de moradia das pacientes. Estes achados vão na mesma direção do presente estudo, e podem ajudar a explicar por que o aumento da cobertura de rastreamento se associa com o aumento da mortalidade em áreas mais pobres.

Para a mortalidade por CCU, há também diminuição dos valores ao longo do tempo para todo o MSP e aparente redução da dispersão nas unidades de análise, mas sem mudanças do padrão espacial. Ou seja, as maiores taxas continuam presentes nas regiões mais periféricas ao longo de todo o período. Este é um resultado igualmente importante; a queda provavelmente se explica pelo aumento da cobertura de rastreamento no município, que propicia detecção e tratamento de lesões precursoras, apesar de ainda ser desigual, havendo menor acesso nas regiões periféricas^27,28^. Essa queda concorda com o perfil de tendência temporal da mortalidade por este câncer no Brasil e suas regiões, exceto na região Norte^25,29^. Em relação às análises estratificadas por faixa etária, a mais jovem (20–39 anos) foi a única que apresentou tendência de aumento, fenômeno que também tem sido observado em países europeus^30^.

Também vale considerar que o valor apresentado referente à taxa global de mortalidade para o CM no município foi mais que o dobro da média mundial, em 2018. Já para o CCU, o valor encontrado foi pouco inferior à média mundial. Destaca-se que essas taxas mundiais estão padronizadas conforme a mesma população padrão, mas sem o filtro para mulheres com 20 anos ou mais de idade^1^.

Algumas limitações deste estudo estão relacionadas ao uso de dados secundários, sujeitos a diferentes graus de qualidade das informações; e às estimativas realizadas para o cálculo das populações dos anos não censitários, uma vez que a precisão dessas estimativas se reduz à medida que se distanciam dos anos censitários. Também são limitações (i) a não consideração dos setores censitários com menos de 50 domicílios particulares permanentes para o cálculo dos valores dos IPVS das AA e (ii) a extrapolação das informações do nível de setor censitário para essas unidades de análise, uma vez que as delimitações censitárias não correspondem às das AA. Esse problema foi contornado utilizando-se ferramentas de análise espacial. Além disso, há a questão da flutuação aleatória, que ocorre devido à existência de áreas com populações pequenas, minimizada pelo uso da metodologia Bayesiana empírica local e do agrupamento em períodos.

Como fortalezas, destaca-se que as estimativas contaram com o auxílio de ferramentas de geoprocessamento, e foi utilizado um amplo período de estudo. Além disso, a qualidade dos registros de mortalidade no MSP resultou em elevada taxa de sucesso da geocodificação (98,9%), indicando, entre outros fatores, um bom preenchimento da declaração de óbito.

O conhecimento dos padrões espaciais e temporais possibilita melhor direcionamento de recursos para prevenção e promoção da saúde nos territórios. Igualmente, levanta questionamentos que podem ser mais bem explorados por outros estudos visando esclarecer melhor as razões para os distintos comportamentos.
